# Neutrophil-sourced TNF in cancer: deciphering an intricate orchestrator of immunosuppressive communication in the tumor microenvironment

**DOI:** 10.1038/s41392-023-01530-4

**Published:** 2023-07-17

**Authors:** Xinyuan Zhao, Ye Lu, Li Cui

**Affiliations:** 1 Stomatological Hospital, School of Stomatology, Southern Medical University, Guangzhou, 510280 Guangdong China; 2 Division of Oral Biology and Medicine, School of Dentistry, University of California, Los Angeles, Los Angeles, 90095 CA USA

**Keywords:** Cancer, Immunology

In a recent *Cancer Discovery* publication, Bianchi et al. unveil a crucial mechanism driving therapeutic resistance in pancreatic ductal adenocarcinoma (PDAC), orchestrated by cell-autonomous Cxcl1 and TNF from polymorphonuclear myeloid-derived suppressor cells (PMN-MDSCs). This insight provides fresh perspectives for surmounting such resistance in PDAC.^1^

PDAC represents a highly aggressive malignancy exhibiting substantial therapeutic resistance, attributable to factors such as intractable genomic aberrations, immunosuppression mediated by myeloid cells, and pro-inflammatory activation of cancer-associated fibroblasts. For instance, spontaneous ferroptosis of PMN-MDSCs within the tumor microenvironment (TME), despite reducing their presence, indirectly suppresses T-cell activity and promotes tumor growth.^2^ Although significant advancements have been made in the field, numerous enigmas persist, particularly concerning the intricate interplay within the TME that fuels therapeutic resistance. A more profound comprehension of these underlying mechanisms is indispensable for devising enhanced treatment strategies and ultimately ameliorating patient prognoses. Bianchi’s groundbreaking research offers an enlightening vantage point on investigating the complex orchestration of interactions among tumor cells, immune cells, and stromal components within the TME.

Through analyzing transcriptomes of human PDAC cell lines, Bianchi et al. discovered that *KRAS-TP53* co-alterations specifically enriched pathways related to neutrophil function and CXCL1 expression. This finding was further validated in preclinical genetic models and patient-derived tumors. Utilizing single-cell RNA sequencing and imaging mass cytometry, the team ascertained that CXCR2, the receptor for CXCL1, was primarily expressed in intratumoral PMN-MDSCs. Furthermore, they observed strong spatial contiguity between CXCL1-expressing tumor islands and CXCR2^+^ PMN-MDSCs, with CD8^+^ T cells being conspicuously absent from these areas.

The researchers also uncovered that the mutational cooperativity of cell-autonomous *KRAS*^*G12D*^ and *TP53*^*R175H*^ increased CXCL1 production *via* CREB-dependent mechanisms. Inhibiting CREB led to a significant decrease in CXCL1 expression and secretion in both human and murine PDAC models. To investigate Cxcl1’s role in T-cell exclusion, the study employed CRISPR-Cas9 edited *K-ras*^*G12D/+*^*;Trp53*^*R172H/+*^*;Pdx-1*^*Cre/+*^ (KPC) tumor cells. The results revealed that Cxcl1 silencing contributed to reduced tumor growth and improved survival. Moreover, enriched immunoregulatory signaling and increased CD4^+^ and CD8^+^ T cells were observed in Cxcl1-silenced tumors, suggesting that Cxcl1 silencing overcomes T-cell exclusion and suppresses tumor growth through a CD8^+^ T-cell mechanism.

The authors then probed the impact of Cxcl1 silencing on MDSC trafficking dynamics and T-cell suppression using a novel in vivo adoptive transfer system. They found that reprogramming of PMN-MDSCs generated in Cxcl1-silenced tumor-bearing hosts disrupted their migratory potential and reduced immunosuppressive markers. This reprogramming of MDSC function could contribute to the improvement of T-cell exclusion and antitumor immunity in Cxcl1-silenced tumors. RNA sequencing identified enrichment in MAPK pathway signaling and Tnf as the top upstream regulator of differentially expressed MDSC transcriptomes. They established a signaling link between CXCR2 ligation, MAPK pathway activation, and Tnf production in PMN-MDSCs, with the dependency of MDSC-intrinsic TNF on CXCR2-MAPK signaling validated in vivo. The study exposed a previously unrecognized paradox where inhibiting Cxcl1-CXCR2 engagement invigorates T-cell activation while dampening tumor-wide TNF signaling, predominantly through disruption of MDSC-restricted TNF.

Shifting focus to the impact of MDSC-derived TNF in the PDAC tumor microenvironment, the study unveiled its crucial role in immunoregulatory circuitries and stromal inflammation. The experiments demonstrated that transmembrane TNF-TNFR2 signaling upregulated the expression of Cxcl1 in tumor cells and cancer-associated fibroblasts (CAFs), leading to increased pro-inflammatory signaling. Etanercept treatment, a TNFR2 inhibitor, considerably decreased Cxcl1 expression and suppressed neutrophil trafficking dynamics in adoptive transfer experiments. Additionally, etanercept preconditioning rescued T-cell IFN-γ release suppression and enhanced intratumoral T-cell trafficking. This treatment also resulted in a significant decrease in PMN-MDSCs and M2-like macrophages. Inhibition of transmembrane TNF-TNFR2 impaired the IL-6/STAT3 signaling pathway in the tumor and diminished the iCAF:myCAF ratio, ultimately attenuating the inflammatory stromal components and fibrotic collagen deposition. Combining etanercept with gemcitabine + paclitaxel chemotherapy nearly doubled the median survival in orthotopic KPC models and reduced metastatic outgrowth compared to single treatments, without additional toxicity. These findings emphasize the potential of TNFR2 inhibition for mitigating stromal inflammation, reducing Cxcl1 expression, and enhancing PDAC’s chemosensitivity, which underscores its therapeutic value.

**Fig. 1:**
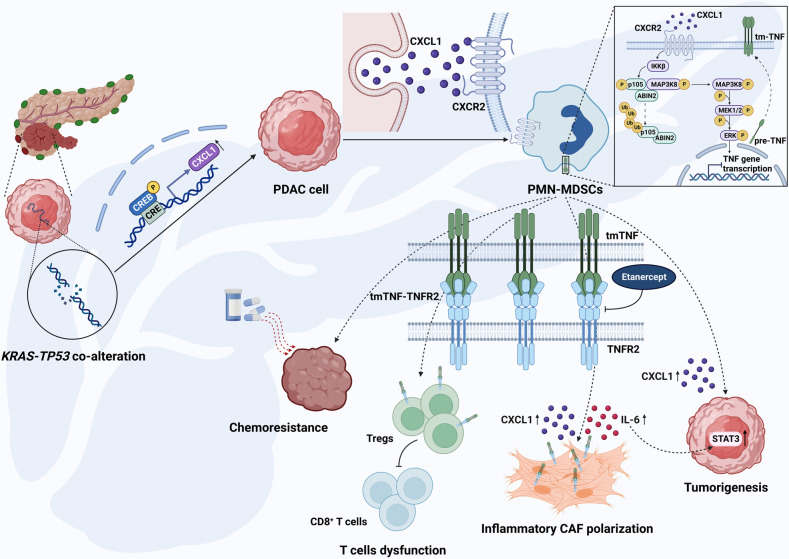
Cell-autonomous Cxcl1 orchestrates T-cell restriction through CXCR2-expressing PMN-MDSC interaction and PMN-MDSC-derived TNF-driven stromal inflammation, immune tolerance, and therapeutic resistance in PDAC. Co-alterations of *KRAS-TP53* transcriptionally enhance CXCL1 expression *via* a CREB-dependent mechanism in PDAC cells, facilitating tumor cell interaction with CXCR2^+^ PMN-MDSCs, which in turn activates the CXCR2-MAPK-TNF signaling cascade in PMN-MDSCs. Neutrophil-derived TNF exacerbates Treg dysfunction in PDAC through transmembrane TNF-TNFR2 signaling, ultimately leading to the suppression of CD8^+^ T-cell activity and hindering the immune response. Concurrently, transmembrane TNF-TNFR2 interactions substantially increase the iCAF:myCAF ratio and amplify IL-6/STAT3 signaling in the tumor, promoting stromal inflammation and therapeutic resistance. Notably, PMN-MDSCs significantly elevate Cxcl1 expression in both PDAC cells and CAFs. Targeting TNFR2 disrupts this intricate network, enhancing chemotherapy sensitivity in vivo. Created with BioRender.com

In summary, Bianchi and colleagues demonstrates that cell-autonomous Cxcl1, regulated by *KRAS-TP53* genomic co-alteration in PDAC, mediates T-cell restriction through interactions with CXCR2^+^ neutrophilic MDSCs, and that TNF signaling in cancer cell-neutrophil crosstalk drives stromal inflammation, immune tolerance, and therapeutic resistance (Fig. 1). However, the study presents several areas for further exploration. Primarily, the impact of transmembrane TNF derived from PMN-MDSCs on immune cells, beyond tumor-infiltrating CD8^+^ T cells, remains nebulous. Additionally, the underpinning mechanism of CD8^+^ T-cell exclusion by PMN-MDSCs is yet to be elucidated. Furthermore, residual CAF-Il6 expression, notwithstanding transmembrane TNF-TNFR2 inhibition in MDSC-CAF co-cultures, suggests additional routes by which neutrophil-derived signals could influence iCAF polarization. Despite these limitations, the insights gained from this study offer valuable directions for future research and have important implications for the development of new therapeutic strategies.

The identification of neutrophils as a significant source of TNF in human and murine PDAC is a critical discovery that sheds new light on the intricate interplay between the immune system and cancer. Overexpression of TNF is a prevalent hallmark in various cancers,^3^ emphasizing the need to determine if this discovery is exclusive to PDAC or a consistent phenomenon spanning a wide array of malignancies. In addition, certain cytokines in the TME were traditionally believed to be generated by specific cell types or stromal components, but it is important to note that other cell types, including previously unrecognized ones, may also produce these cytokines in response to TME-induced changes. Consequently, it is imperative to adopt a more comprehensive and meticulous approach to accurately determine the cellular sources of cytokines in the TME, which may lead to a deeper understanding of the mechanisms underlying cancer progression and inform the development of novel therapeutic strategies.

TNFR2 antagonism has been widely accepted as a strategy to eliminate tumor-residing Tregs and activate Teff expansion, making it a potentially attractive combination therapy.^4,5^ Additionally, TNFR2 has been reported to be highly expressed in some tumor cells, suggesting that targeting TNFR2 might also directly kill TNFR2-expressing tumor cells.^4,5^ However, the detailed role of TNFR2 in PDAC cells remains to be fully investigated. Bianchi et al. have reported that TNFR2 expression was induced in CAFs by co-culture with PMN-MDSCs, suggesting that targeting TNFR2 also alter the malignant behaviors of CAFs and further reshape the tumor microenvironment. The consequences of targeting TNFR2 on various TNFR2-expressing cell types within the TME merit additional exploration. These multiple potential benefits of TNFR2 targeting suggest that it might prove to be a promising approach for cancer therapy. Nevertheless, while preclinical studies have demonstrated efficacy, the efficacy and safety of TNFR2-targeting therapies need to be confirmed through human clinical trials.
